# Supplementary material for: Enhanced Stability of Li-RHC Embedded in an Adaptive TPX™ Polymer Scaffold
Source: Materials (Basel). 2020 Feb 22;13(4):991. doi: 10.3390/ma13040991 (PMC7078616; doi:10.3390/ma13040991)
Supplement: Supplementary file 1 [file materials-13-00991-s001.pdf]

Supporting information

Enhanced Stability of Li-RHC Embedded in an Adaptive TPX™ Polymer Scaffold

Thi Thu Le [1,*], Claudio Pistidda [1,*], Clarissa Abetz [2], Prokopios Georgopanos [2], Sebastiano Garroni [3], Giovanni Capurso [1], Chiara Milanese [4], Julián Puszkiel [1,5], Martin Dornheim [1], Volker Abetz [2,6], Thomas Klassen [1,7]

1. Institute of Materials Research, Materials Technology, Helmholtz-Zentrum Geesthacht GmbH, Max-Planck-Strasse 1, D-21502 Geesthacht, Schleswig-Holstein, Germany
2. Institute of Polymer Research, Helmholtz-Zentrum Geesthacht, Max-Planck-Str. 1, 21502 Geesthacht, Germany
3. Department of Chemistry and Pharmacy and INSTM, University of Sassari, via Vienna 2, 07100. Sassari, Italy
4. Pavia H_2 Lab, C.S.G.I. & Department of Chemistry, Physical Chemistry Section, University of Pavia, Italy
5. Department of Physical Chemistry of Materials, Consejo Nacional de Investigaciones Científicas y Técnicas (CONICET) and Centro Atómico Bariloche, Av. Bustillo km 9500 S.C. de Bariloche, Argentina
6. Institute of Physical Chemistry, Universität of Hamburg, Martin-Luther-King-Platz 6, 20146 Hamburg, Germany
7. Helmut Schmidt University, University of the Federal Armed Forces Hamburg, 22043 Hamburg, Germany

* Correspondence: thi.le@hzg.de (T.T.L.); Tel.: +49-4152-87-2687 (T.T.L.); claudio.pistidda@hzg.de (C.P.); Tel: +49-4152-87-2644 (C.P.)

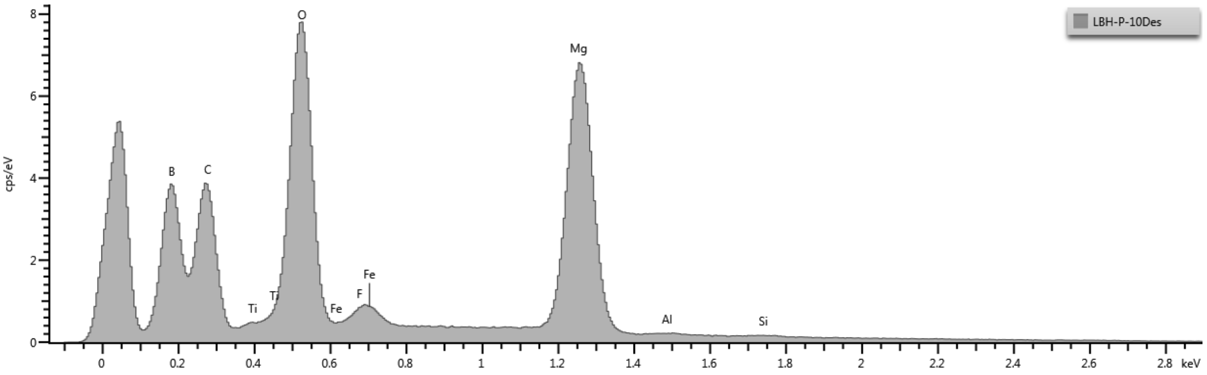

Figure S1. EDX spectrum

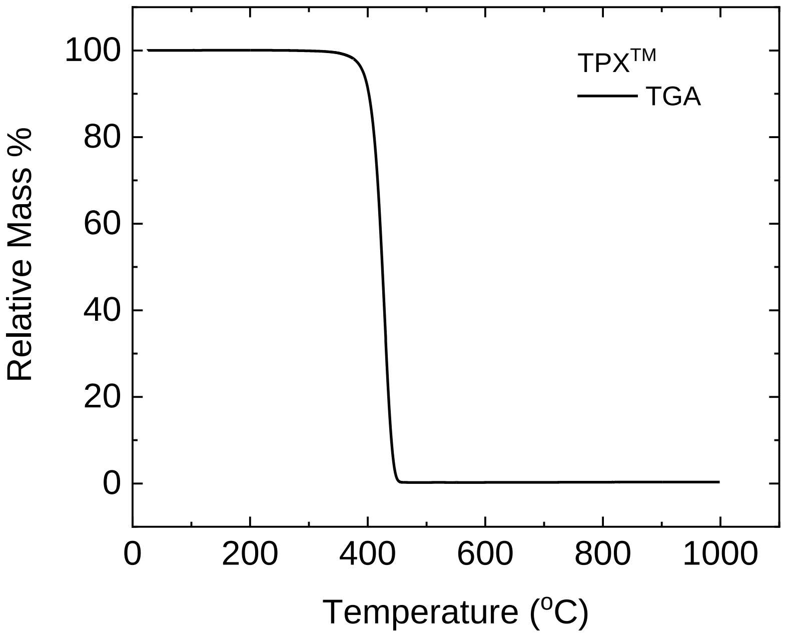

Figure S2. Thermogravimetric analysis of the TPXTM polymer. Heating rate 10 K/min under argon atmosphere.

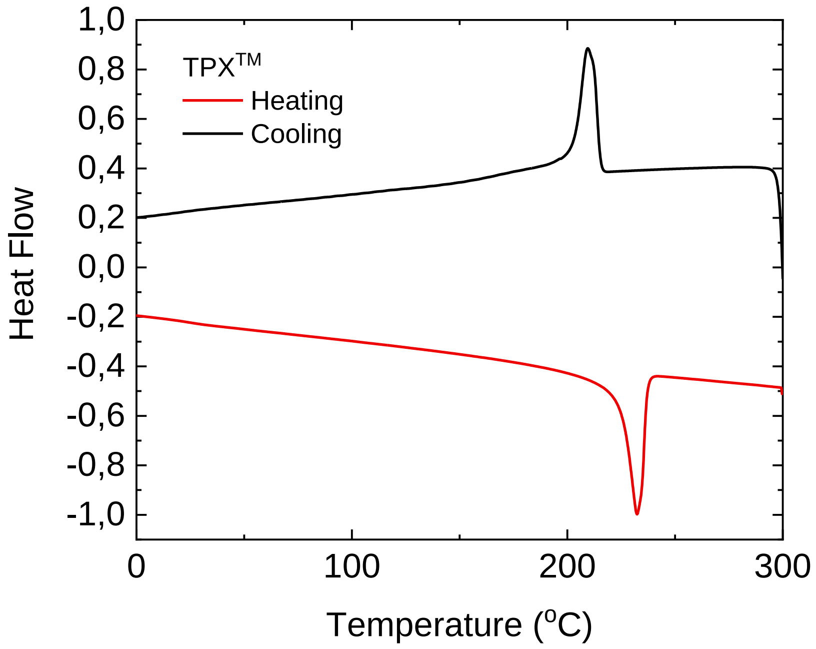

Figure S3. DSC Heating and cooling traces (2nd cycle) for the TPX™ polymer. Heating rate 10 K/min under nitrogen atmosphere.
